# Titanium implants and silent inflammation in jawbone—a critical interplay of dissolved titanium particles and cytokines TNF-α and RANTES/CCL5 on overall health?

**DOI:** 10.1007/s13167-018-0138-6

**Published:** 2018-06-08

**Authors:** Johann Lechner, Sammy Noumbissi, Volker von Baehr

**Affiliations:** 1 Clinic for Integrative Dentistry, Grünwalder Str. 10A, Munich, Germany; 2 Miles of Smiles Implant Dentistry, 801 Wayne Ave no. G200, Silver Spring, USA; 3 Institute for Medical Diagnostics in MVZ GbR, Nicolaistr. 22, 12247 Berlin, Germany

**Keywords:** Dental titanium implants, TNF-α, RANTES/CCL5, Fatty degenerative osteonecrosis of the jawbone, Immune system disorders, Silent inflammation, Preventive medicine

## Abstract

**Background and introduction:**

It is a well-known fact that titanium particles deriving from dental titanium implants (DTI) dissolve into the surrounding bone. Although titanium (TI) is regarded as a compatible implant material, increasing concern is coming up that the dissolved titanium particles induce inflammatory reactions around the implant. Specifically, the inflammatory cytokine tumor necrosis factor-alpha (TNF-α) is expressed in the adjacent bone. The transition from TNF-α-induced local inflammation following insertion of DTI surgery to a chronic stage of “silent inflammation” could be a neglected cause of unexplained medical conditions.

**Material and methods:**

The signaling pathways involved in the induction of cytokine release were analyzed by multiplex analysis. We examined samples of jawbone (JB) for seven cytokines in two groups: specimens from 14 patients were analyzed in areas of DTI for particle-mediated release of cytokines. Each of the adjacent to DTI tissue samples showed clinically fatty degenerated and osteonecrotic medullary changes in the JB (FDOJ). Specimens from 19 patients were of healthy JB. In five cases, we measured the concentration of dissolved Ti particles by spectrometry**.**

**Results:**

All DTI-FDOJ samples showed RANTES/CCL5 (R/C) as the only extremely overexpressed cytokine. DTI-FDOJ cohort showed a 30-fold mean overexpression of R/C as compared with a control cohort of 19 healthy JB samples. Concentration of dissolved Ti particles in DTI-FDOJ was 30-fold higher than an estimated maximum of 1.000 μg/kg.

**Discussion:**

As R/C is discussed in the literature as a possible contributor to inflammatory diseases, the here-presented research examines the question of whether common DTI may provoke the development of chronic inflammation in the jawbone in an impaired state of healing. Such changes in areas of the JB may lead to hyperactivated signaling pathways of TNF-α induced R/C overexpression, and result in unrecognized sources of silent inflammation. This may contribute to disease patterns like rheumatic arthritis, multiple sclerosis, and other systemic-inflammatory diseases, which is widely discussed in scientific papers.

**Conclusion:**

From a systemic perspective, we recommend that more attention be paid to the cytokine cross-talk that is provoked by dissolved Ti particles from DTI in medicine and dentistry. This may contribute to further development of personalized strategies in preventive medicine.

## Background

Tooth replacement is a long-sought-after and practiced solution in dentistry. In modern dentistry, one of the most established and proven methods is the replacement of lost or missing teeth with dental implants. The most popular and proven implant materials are titanium and titanium alloys which have shown to be very successful in terms of their integration with surrounding tissue. However, removal of the tooth and subsequent implantation submit the jawbone and body to an inflammatory process that, in most cases, leads to uneventful healing and implant stability. Multiple investigators have found that titanium implants can induce inflammation in the surrounding tissue over time, leading to the expression of certain mediators known to cause local and systemic health problems. While acute disease is unavoidable, chronic diseases (cancer, autoimmune diseases, etc.) are the clinical and visible manifestations of a constantly stimulated immune system [1–4]. These triggers lead to the activation of signaling pathways which favor a predisposition to the development of chronic disease. In general, cell communication systems are organized as cascades with sequenced stages [5]. Signaling messengers like cytokines carry instructions and are received by those cells with specific receptors which are able to recognize them. In earlier publications, we defined this chronic inflammatory process as fatty degenerative osteonecrosis in the medullary spaces of the jawbone (FDOJ) [6, 7]. Peri-implant pathology also commonly known as “peri-implantitis” is a widely discussed phenomenon based on immunological susceptibility to TI and macrophage reaction which can ultimately lead to titanium implant (T-IMP) failure [1, 8, 9]. In daily dental practice, the effects of implants on overall health are often overlooked because local problems appear to resolve after the implants are healed and integrated.

## Objectives

This research aims to elucidate the transition from acute trauma during the insertion of dental implants to chronic inflammation (CI) of the jawbone. Herein, we attempt to define the role of cytokines in areas of FDOJ surrounding implants in a cohort of patients with immune system disorders (ISD). We propose the following hypothesis: T-IMPs may be a possible contributor to the development of CI of the jawbone extending beyond the local condition of peri-implantitis. Individuals with specific risk factors may be more susceptible to subsequently developing certain types of systemic diseases (i.e., ISD).

## Material and methods

### Groups of patients examined

Patients with T-IMP (*n* = 14) were selected. This T-IMP group consisted of patients with well-osseointegrated T-IMPs and with clinical symptoms of ISD diagnosed by internal medicine physicians with the following ISD as inclusion criteria: rheumatic arthritis (RA) = 7, neurodegenerative diseases characterized by a progressive loss of neuron function including chronic fatigue syndrome (CFS) and multiple sclerosis (MS) = 3, ovarian cancer (OvC) = 1, and atypical facial pain/trigeminal neuralgia (TrN) = 3. A second mandatory inclusion criterion for the T-IMP group was local diagnosis of FDOJ apically and in areas surrounding T-IMPs. The average age of the T-IMP cohort was 56 years (SD = ± 11.4) and the gender ratio was 13:1 (F/M). All enrolled patients were required to have a panoramic radiograph (2D-OPG), cone beam computed tomography imaging (3D-CBCT), and measurement of the bone density of the jawbone using the transalveolar ultrasound technology (TAU). TAU is a useful tool to establish the presence of FDOJ [10]. Tissue samples from areas of FDOJ identified in the 14 patients comprising the study cohort were collected.

In a healthy control group (*n* = 19), samples of healthy jawbone (HJB) were removed in the form of drill cores during routine dental implantation surgery. Inclusion criteria for this latter group consisted of the following: a 2D-OPG showed no radiological distinctive features and TAU measurements of bone density at the site of implantation were inconspicuous. The age range of the control group was 38–71 years of age with an average age of 54 (SD = ± 12.4) and a gender ratio of 11:8 (F/M). The fact that some patients were undergoing treatment for their diagnosed systemic disease was not an exclusion criterion. The use of bisphosphonate medication, however, was the main exclusion criteria also for this group. This research was done based on data obtained from retrieved tissue samples from patients during routine dental surgery after obtaining their written informed consent. This study was performed as a randomized control trial and statistical analyses were performed using IBM SPSS, version 19 (IBM Corporation, Armonk, NY, USA). All data was presented as a mean ± standard mean error. Data was considered significant where the value was < 0.05.

### Clinical features of fatty degenerative osteonecrosis of the jaw: definition and diagnostic criteria

**Fig. 1:**
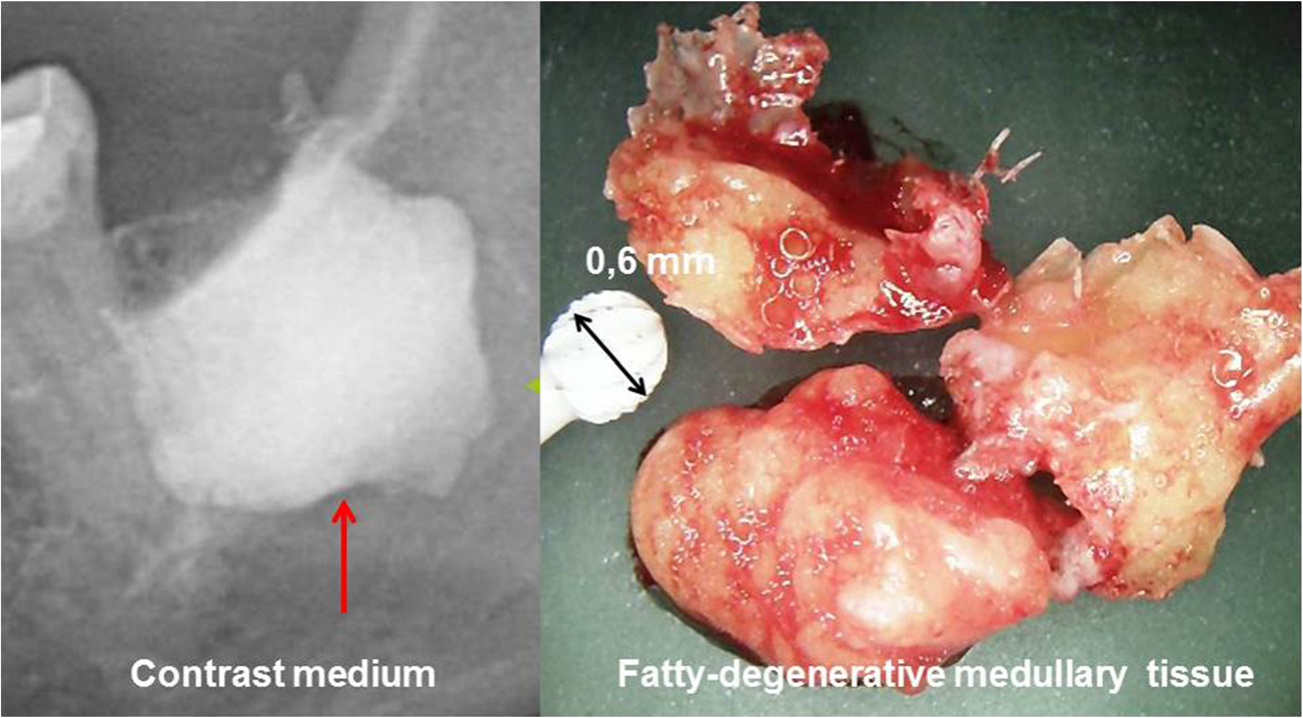
FDOJ sample of fatty and osteolytic degenerated bone marrow (right panel) and contrast medium in FDOJ cavity after curettage (left panel)

FDOJ is a lesion similar to that found in long bones, also primarily defined as “bone marrow edema” and “chronic non-suppurative osteomyelitis” [11]. The softening of the bone marrow in FDOJ is very distinct, such that the marrow space may be suctioned out or curetted once the cortical bone is removed. These hollow spaces, also known as “cavitations,” are filled with fatty degenerated adipocytes which have undergone dystrophic changes accompanied by demyelination of the bony sheath of the inferior alveolar nerve. All 14 FDOJ samples from the ISD group presented clinically and macroscopically as fatty lumps. The image in Fig. 1 shows a specimen of predominantly fatty transformation of the jawbone. The extent of the FDOJ lesion in the jawbone is indicated in the X-ray image with contrast medium.

### Pathohistological examination

In parallel with the cytokine analysis, each FDOJ sample was examined histopathologically (Institute for Pathology and Cytology; Drs. Zwicknagel/Assmus 85635 Freising, Germany).

### Dissolved titanium particles in the jawbone

Following multiple reports in the literature concerning dissolved TI particles in the surrounding bone [2, 12–15], we analyzed five of the 14 jawbone samples from the group with FDOJ-T-IMP for dissolved TI levels. (Medizinisches Labor Bremen 28357 Bremen, Germany.)

### Sampling of FDOJ-T-IMP tissue

**Fig. 2:**
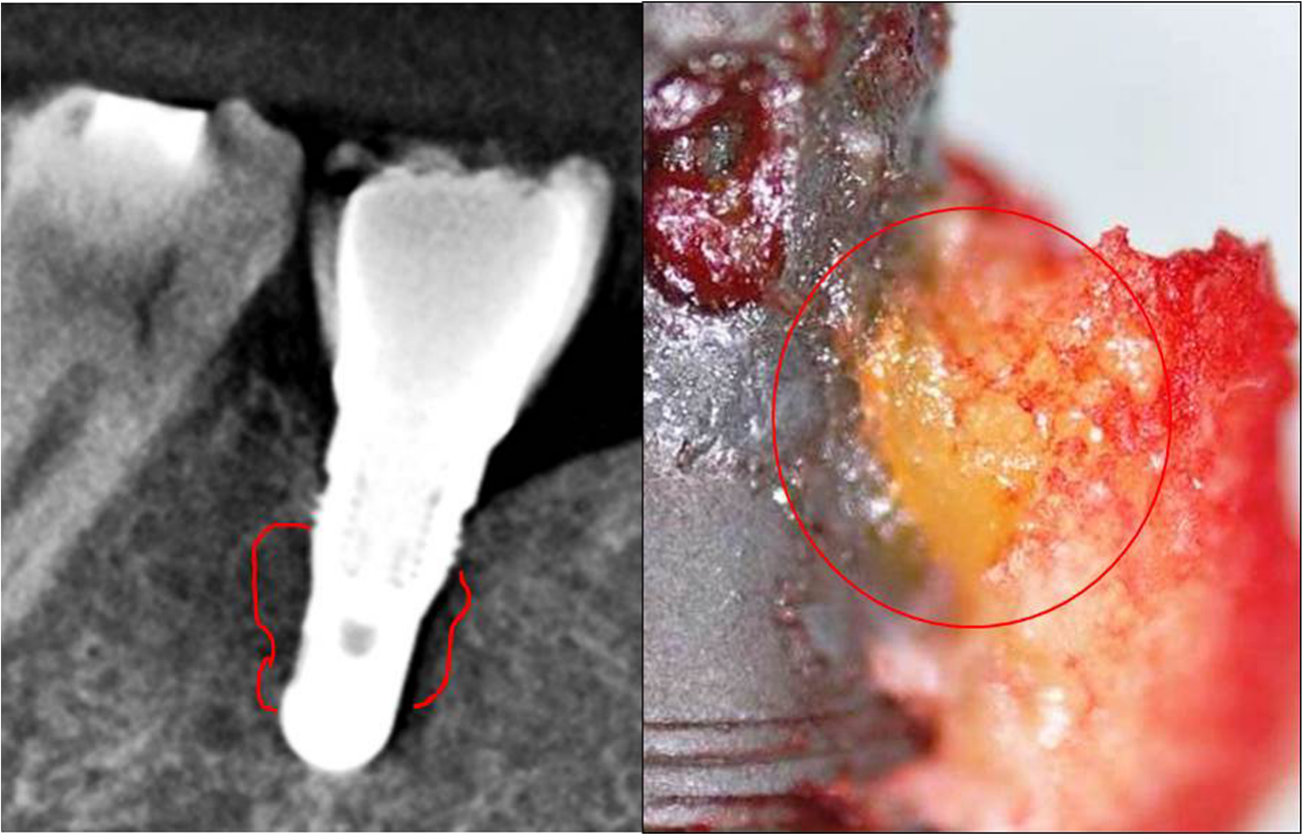
Titanium implant area 46 as shown in 3D-DVT. Fatty degenerated tissue attached directly to the titanium implant

Current treatment of FDOJ lesions consists of curettage of the bony cavity [16]. To discern the cytokine patterns found in jawbone of patients from the corresponding author’s dental practice, 14 patients with diagnosed FDOJ in T-IMP sites had surgery on the affected area of the jaw, including the removal of existing T-IMPs. After local anesthesia, the mucoperiosteal flap was raised and the cortical layer removed. All patients displayed FDOJ in the bone marrow adjacent to neighboring T-IMPs which was similar to FDOJ samples as described previously in the literature [17, 18] and in Fig. 1 (see above). FDOJ samples with a volume of up to 0.5 cm^3^ were stored in a dry, sterile 2-mL collection vial (Sarstedt AG and Co, Nümbrecht, Germany) that was sealed and frozen at − 20 °C. In these 14 patients, surgery was performed during explantation of T-IMPs from adjacent spongy bone marrow. FDOJ tissue directly attached to a T-IMP was investigated and the cytokine profiles were evaluated. An example is shown in Fig. 2 where the corresponding cone beam 3D image displays no, or only minor, abnormalities in contrast to the significant area of yellowish, discolored, and softened cancellous bone directly attached to the T-IMP surface.

### Analysis of necrotic tissue samples and measurement of cytokines

At the Institute for Medical Diagnostics, Nikolaistr. 22, D-12247 Berlin (inspected by DAKKS, Deutsche Akkreditierungsstelle GmbH, and accredited to DIN EN ISO/IEC 17025:2005 and DIN EN ISO 15189:2007), the FDOJ samples were homogenized by centrifugation in 200 μL of cold protease inhibitor buffer (Complete Mini Protease Inhibitor Cocktail; Roche Diagnostics GmbH, Penzberg, Germany). The homogenate was centrifuged for 15 min at 13,400 rpm. Following this, the supernatant was collected and centrifuged for a further 25 min at 13,400 rpm. In the 14 supernatants of tissue homogenate, levels of the following cytokines were measured: RANTES, FGF-2, interleukin (IL)-1 receptor antagonist (ra), IL-6, IL-8, monocyte chemotactic protein-1 (MCP1), and tumor necrosis factor-alpha (TNF-α). Measurement was performed using the Human Cytokine/Chemokine Panel I (MPXHCYTO-60K; Merck KGaA, Darmstadt, Germany) according to the manufacturer’s instructions and analyzed using the Luminex® 200™ with xPonent® Software (Luminex Co, Austin, TX, USA).

## Results

### Histology of tissue surrounding titanium implants

**Fig. 3:**
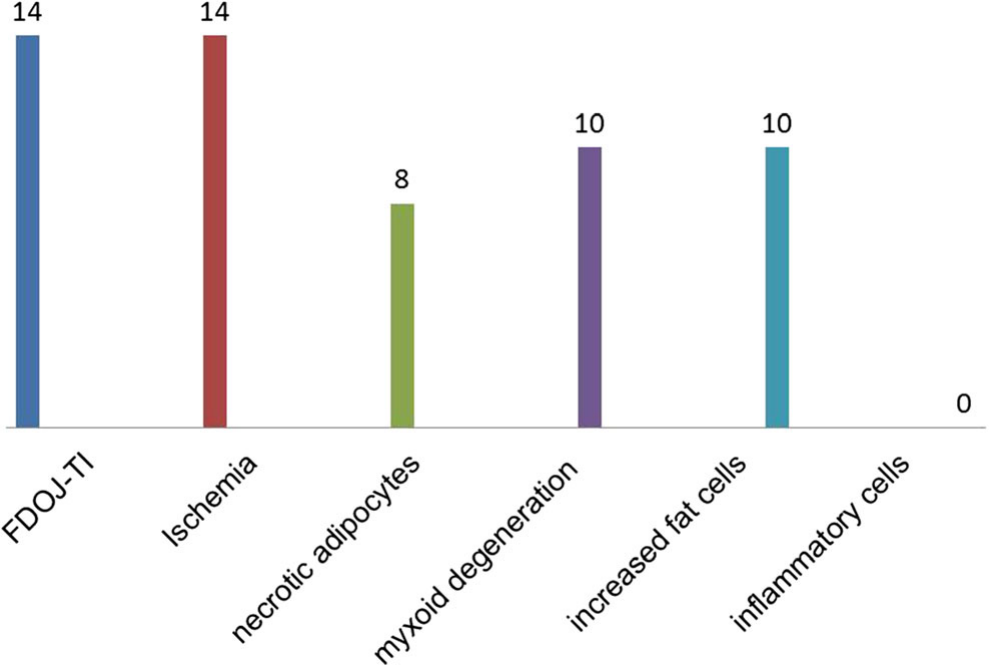
Histological findings from FDOJ samples surrounding titanium implants (FDOJ-T-IMP/*n* = 14)

Histological findings from the 14 samples of FDOJ areas immediately adjacent to a T-IMP were made. Most notably, the tissue exhibited ischemia or trophic disorder with myxoid, fibrillar, or necrotic transformation of the medullary fat cells. Typical inflammation was not found in any case. The number of fat cells was consistently and significantly increased. Typical signs of inflammation and inflammatory cell responses in particular were absent. The fatty degenerative and osteolytic characteristics are the result of insufficient metabolic supply in an ischemic environment. Histologic examination of the curetted tissue indicated ischemia (*n* = 14), necrotic adipocytes (*n* = 8), myxoid degeneration (*n* = 10), and increased fat cells (*n* = 10). However, inflammatory cells were not found in the FDOJ samples. Figure 3 shows the pathohistological findings from the 14 FDOJ samples from those areas surrounding or adjacent to explanted T-IMPs.

### Amount of dissolution of titanium in the jawbone

**Fig. 4:**
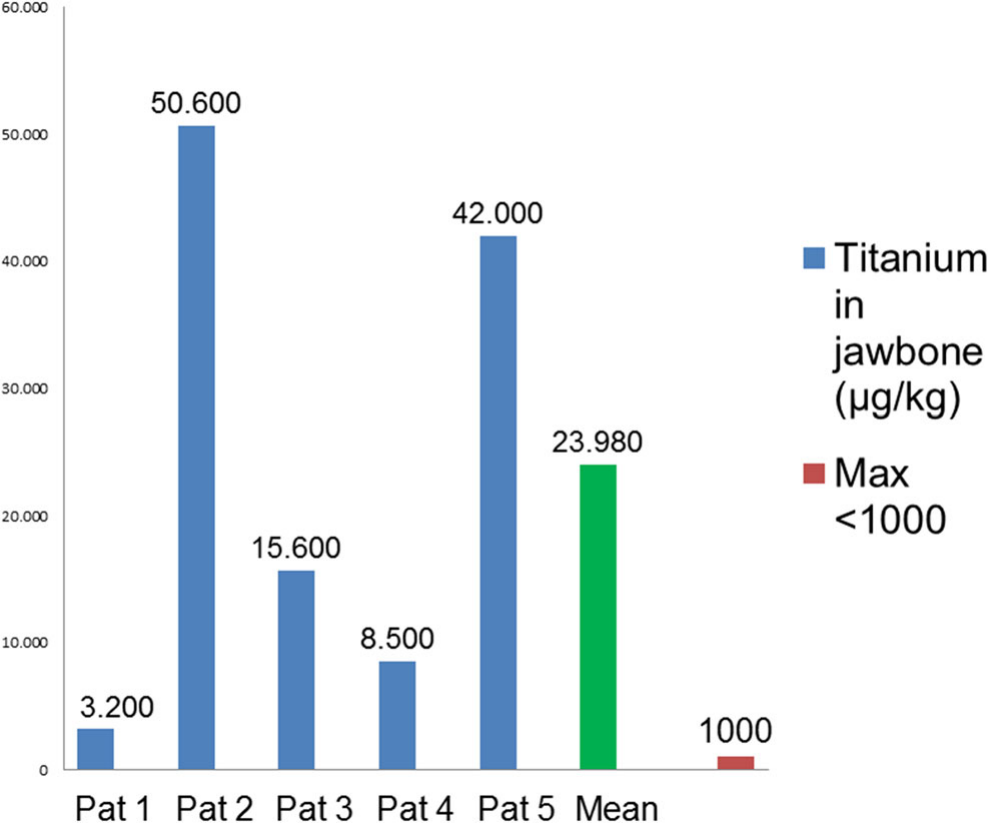
Distribution of dissolved titanium in jawbone surrounding titanium implants in five FDOJ cases

Figure 4 shows the amount of dissolved TI in five of the 14 jawbone FDOJ-T-IMP samples ranged from 3200 to 50,600 μg/kg with a medium value of 24,200 μg/kg (± 20,029 SDEV). As we were unable to find an average maximum content of dissolved TI which is regarded as biocompatible and acceptable in the literature, we defined the maximum dissolved TI in healthy bone as 1000 μg/kg body weight which is fourfold higher than the accepted level of all other heavy metals as described in the relevant literature (< 250 μg/kg).

### Results of cytokine analysis in FDOJ samples

**Fig. 5:**
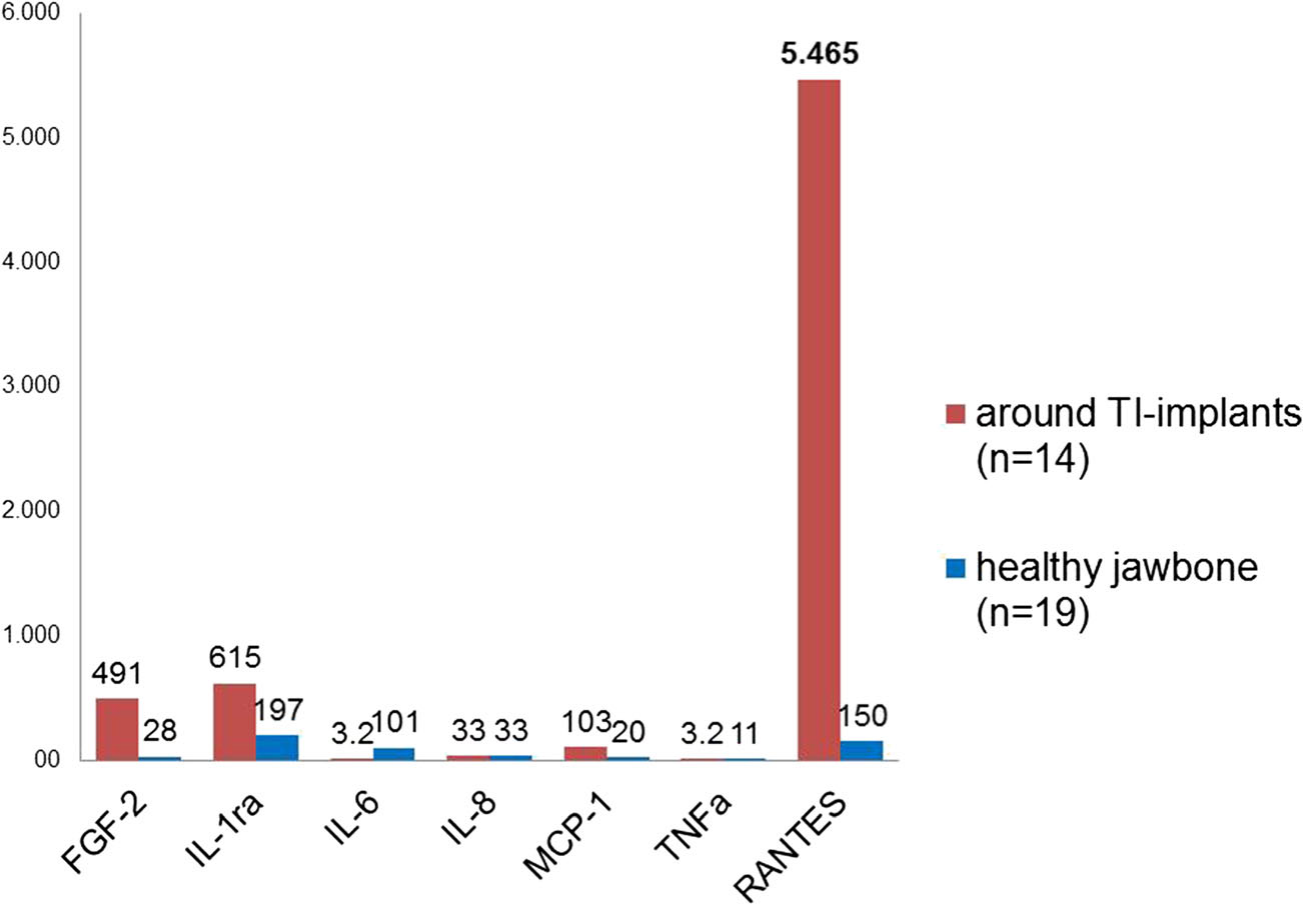
Analysis of seven cytokines in the FDOJ-T-IMP cohort (*n* = 14) (red columns), compared to the healthy jawbone group (*n* = 19) (blue columns)

**Fig. 6:**
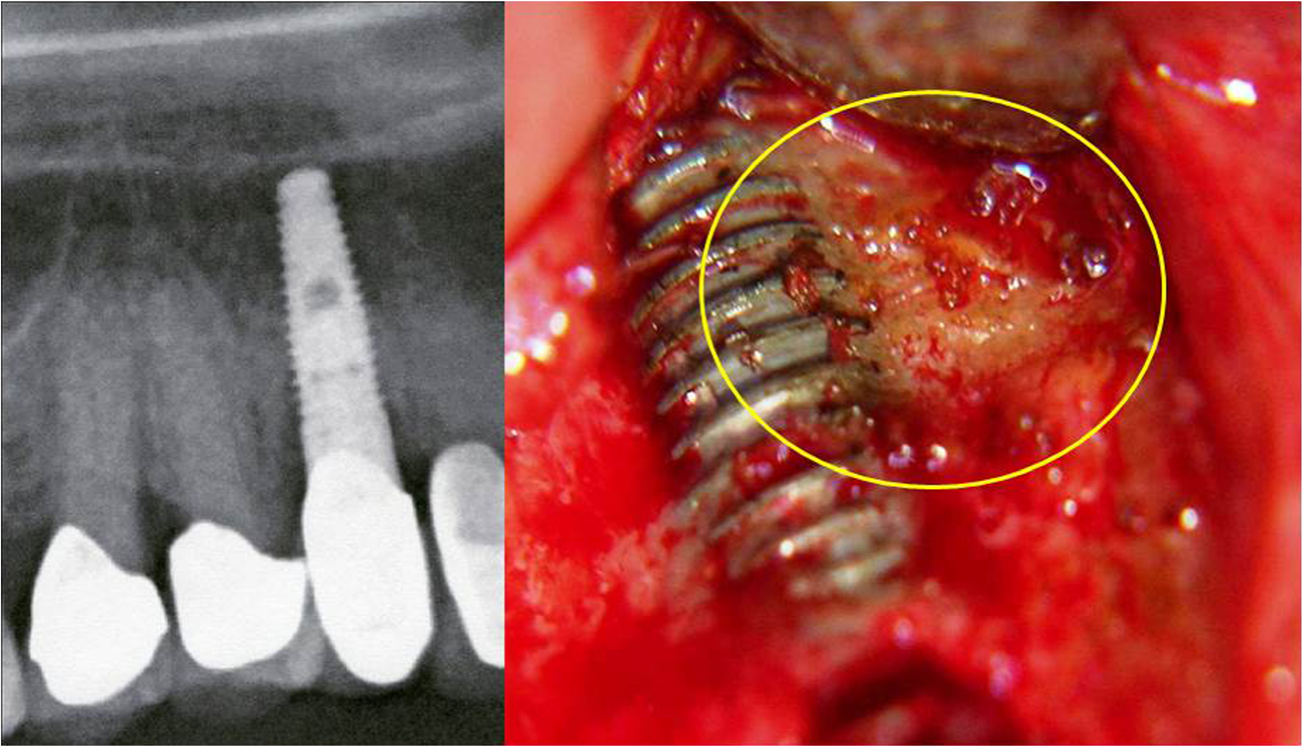
Attachment of fatty degenerative bone (FDOJ) to titanium implant (area 13). X-ray does not indicate inflammatory bone loss or significant peri-implantitis

As we showed in several previous publications [6, 7], the defining characteristic of FDOJ regions is the overexpression of the proinflammatory messenger RANTES, which is regulated on activation by normal T cell expression and secretions also known as Chemokine C-C motif ligand 5 (CCL5). The mean values of 19 samples of HJB were as follows (pg/mL): FGF-2, 27.6; IL-1ra, 196.5; IL-6, 101.0; IL-8, 7.5; MCP-1, 20.3; TNF-α, 11.0; RANTES/CCL5 (R/C), 149.9, (see Fig. 5, blue columns). Values for HJB from healthy patients were not available in the literature for comparison. The results of the multiplex analysis of the seven cytokines in the FDOJ-T-IMP cohort (*n* = 14) are shown in Fig. 5 in red columns: FDOJ samples from jawbone surrounding or adjacent to T-IMP show signs of elevated inflammation with an average R/C value of 5.465.0 (pg/ml) (SDEV = ± 2778) compared to the samples of healthy jawbone from a randomized, controlled group with an average value of 150.0 (pg/ml). All other cytokines, except FGF-2 (fibroblast growth factor 2) and IL-1ra (interleukin 1 receptor antagonist), were not derailed. Figure 6 presents an example of the type of morphology of FDOJ samples removed from T-IMP adjacent areas which were collected and subsequently analyzed for seven cytokines.

## Discussion

FDOJ is similar to silent or subclinical inflammation without the typical signs of acute inflammation. In CI, the local production of inflammatory cytokines, such as TNF-α and IL-1/6, overwhelms regulatory and compensating mechanisms contributing to the formation of FDOJ in the bone marrow. This phenomenon of an intramedullary source of R/C overexpression appears to be more widespread than dentists and physicians previously presumed. The surgical debridement of FDOJ areas, however, may halt the induction of R/C signaling pathways and thus possibly inhibit the progression of associated symptoms [7, 19].

### The problem of diagnosing FDOJ lesions by X-ray

Why is the phenomenon described here such an enigma within the field of dentistry? In previous research, we demonstrated the non-visibility and lack of obvious radiographic signs of FDOJ which make it difficult to obtain an accurate diagnosis using common dental radiographs [20]. As a result, the existence of FDOJ and its systemic implications is largely neglected in mainstream dentistry today. While conventional X-ray techniques are limited in their ability to reveal the actual extent and location of FDOJ, other means of identifying the presence of FDOJ are available. To aid the practitioner in diagnosing the bone marrow softening occurring within FDOJ lesions, a computer-assisted transalveolar ultrasound (TAU) device is available [21]. TAU precisely images and identifies cavitational porosity in the jawbone and has proven to be significantly superior to radiology for the detection of microscopically confirmed FDOJ. In numerous publications, the efficiency and reliability of TAU in the diagnosis and imaging of FDOJ has been presented [22]. Due to these diagnostic difficulties, FDOJ is underdiagnosed by dentists in general and, consequently, also by physicians in ISD cases. It should be noted that while X-rays in most cases fail to diagnose FDOJ, the overexpression of proinflammatory signaling pathways in corresponding FDOJ areas is present and detectable as shown in Figs. 5 and 6. This phenomenon is crucial in the discussion about “silent inflammation.”

### Histology of FDOJ-T-IMP samples indicates no acute inflammation

The lack of any inflammatory cells in the FDOJ samples confirms an inflammation-free progression of bone degeneration and the absence of granulation tissue in FDOJ [16, 23]. In summary, the pathohistological findings clearly demonstrate that histological characteristics in FDOJ-T-IMP are not caused by an osteitic or osteomyelitic process with typical symptoms like swelling, local inflammation, and peri-implantitis. FDOJ should not be confused with any form of osteomyelitis which is characterized by a dramatic increase in inflammatory cells. The histology of the bone from all 14 FDOJ-T-IMP samples, as shown in Figs. 2 and 6, indicates that ischemia is the most prominent feature, which is referred to as a “trophic disorder” in other reports found in the literature, rather than any signs of specific or florid inflammation.

### Titanium dissolution in jawbone and TNF-α expression

TI particles, which are unable to be detected, may dissolve and, under certain conditions, induce immunological reactions in the body and liberate systemic messengers. TI is not as stable as it is often claimed. A study presented by Nakashima et al. [24] elucidated the mechanisms of macrophage activation by TI particles from implant materials and identified the cytokine-bound signaling activated by metal alloy implants via released particles. Macrophages of patients were exposed to particles of TI alloys which were taken from the connective tissue surrounding hip implants. The results of the researchers are not particularly indicative of a “fundamental and natural compatibility of TI as an implant material”: exposure of macrophages to TI alloy particles in vitro over a period of 48 h resulted in a 40-fold increase in the release of TNF-α and a sevenfold increase in the release of interleukin-6.

### Case study of high titanium dissolution in jawbone

**Fig. 7:**
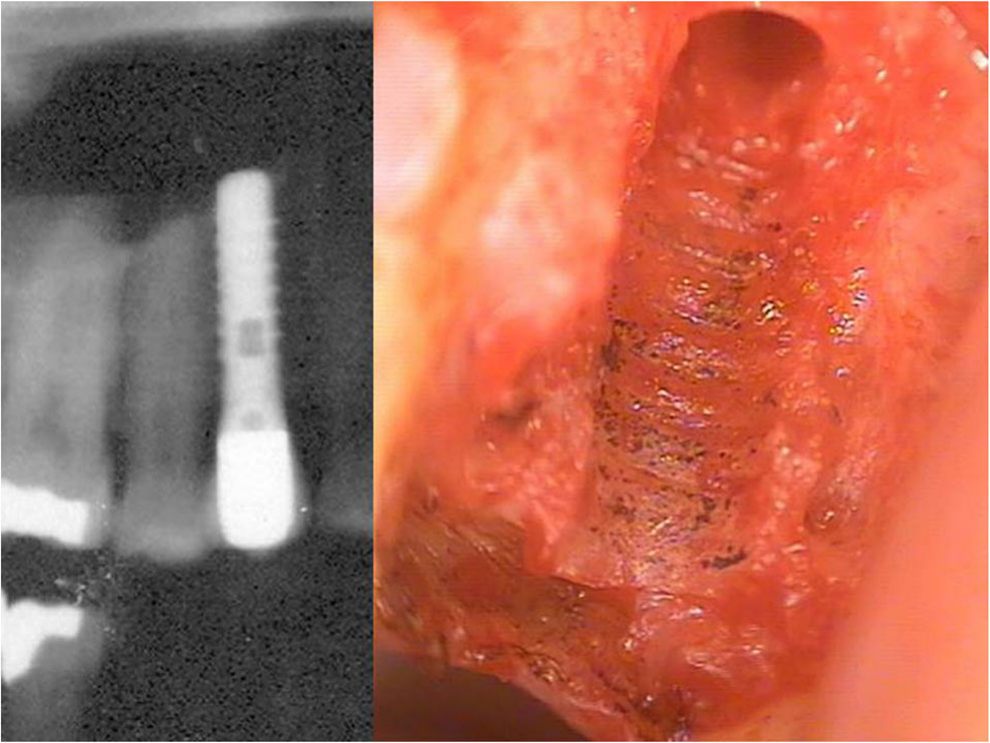
X-ray image prior to removal of titanium implant from area 15 (left panel). Alveolar bone with precipitation of titanium particles (right panel)

**Fig. 8:**
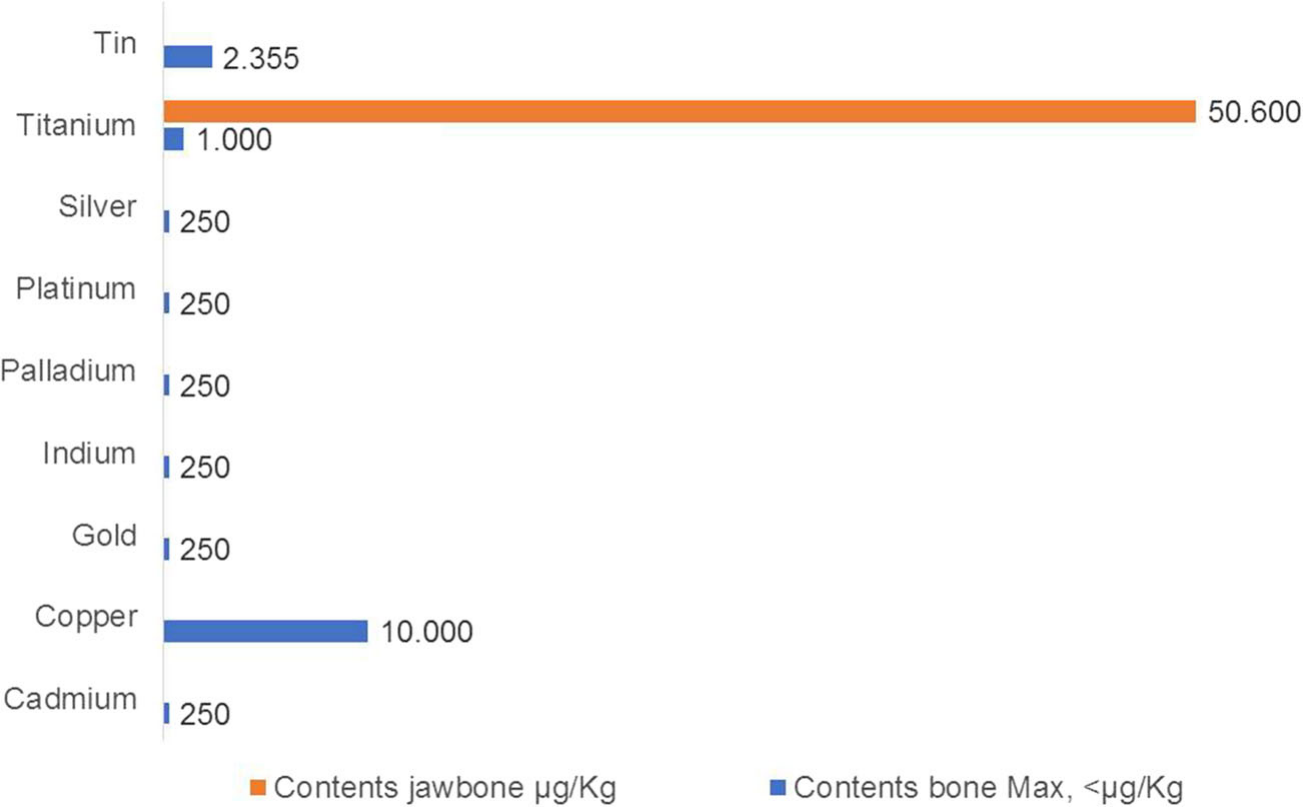
Titanium content in the jawbone (area 15) as determined by spectral analysis

**Fig. 9:**
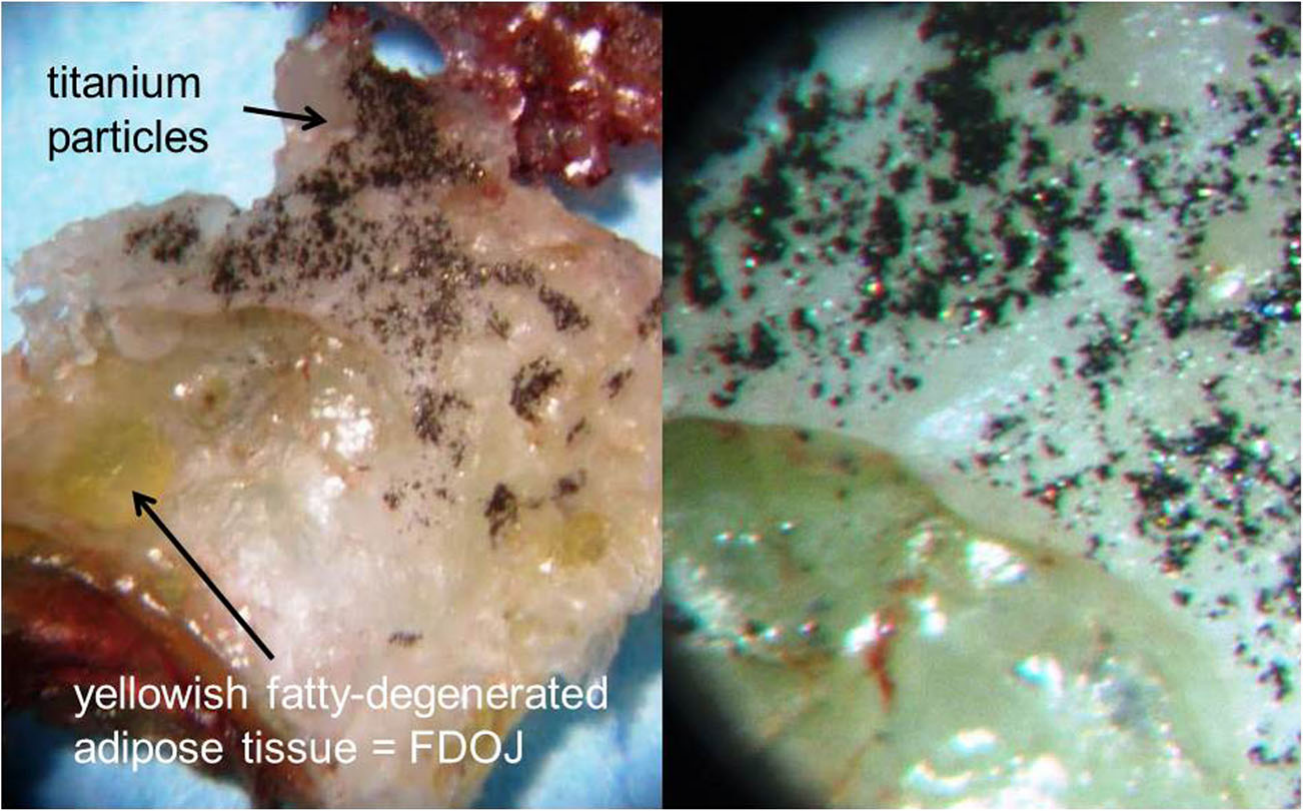
Image of jawbone containing titanium particles from removed implant (left panel). Fourfold microscopic enlargement of titanium particles (right panel)

A case study of a patient from the clinic of one of the authors highlights the problem of the deceptive reassurance that radiographs appear to provide in relation to the fact that there is possible release of TI particles to the per-implant environment. The X-ray of a T-IMP in area 15 showed no suspicious reactions and could be viewed radiographically and mechanically as a successful implant based on the established criteria. In contrast, the image in Fig. 9 shows, after the explantation of the T-IMP, a blackish metallic precipitate in the alveolus along the bony grooves formed by the threads of the removed T-IMP. When the jawbone containing this precipitate was analyzed by spectral analysis for heavy metal contents (Figs. 7, 8, and 9), the value for TI increased by a factor of 50 with an assumed limit value of < 1000 μg per kilogram body weight. The osseous tissue surrounding the implant thus contained 50-fold times the acceptable limit of TI. Figure 9 shows both in detail (left panel) and also magnified fourfold (right panel) the dissolved TI particles found in the bone, including the fatty degenerative adipocytes in the FDOJ area.

### Sensitization of immune system by titanium implants

TNF-α is a proinflammatory cytokine and is at the beginning of almost every immune response based on individual sensitization to TI. Thus, TNF-α plays a key role in the failure of many implants in the case of genetic incompatibility [1, 8, 9]. In addition to the particles released from implant wear and fatigue, “titanium sensitization “ is also the result of increased proinflammatory reactivity of non-specific immune cells (tissue macrophages, monocytes), which in some patients occurs after contact with particulate debris, i.e., TI particles.

It is known that such particles (diameter 1–10 μm) are consistently released into the environment surrounding implants as mechanical abrasion, chemical, bacterial, and galvanic corrosion [25] may induce inflammation which produces in the corresponding hyperinflammatory conditions [26]. Sterner et al. investigated the effects of clinically relevant aluminum ceramic, zirconium ceramic, and titanium particles of varying size and concentration on TNF release in a human macrophage system [3]. The results confirm our experience and reservations: in a direct comparison of Al_2_0_3_ and TI particles of the same size and concentration, TI stimulated significantly higher TNF-α distributions. ZrO_2_ did not induce significant TNF-α secretion.

In the “Amount of dissolution of titanium in the jawbone” section, we demonstrated the solubility of TI particles in the jawbone with reference to several images: following contact with such TI particles, tissue macrophages release proinflammatory cytokines as part of an inflammatory reaction [26]. The extent of the release of proinflammatory cytokines is determined by polymorphisms in the genes of the respective cytokines and thus varies individually. Jacobi-Gresser et al. found that patients with implant loss or peri-implantitis showed significantly more pronounced genetic predisposition to inflammation as well as markedly elevated positive immunological tests results with overactivation of TNF-α and IL-1b [9]. The most important proinflammatory effects of IL-1 and TNF-α are as follows: stimulation and recruitment of other immune cells such as T cells, B cells, and macrophages; activation of the vascular endothelium; central nervous system induction of fever, anorexia, and somnolence; and osteoclast activation which leads to bone resorption. Thus, the two cytokines TNF-α and IL1 are the key mediators of a local but also systemic inflammatory response [27]. The extent to which the proinflammatory cytokines are released after contact with TI oxide particles differs individually. The basis for supernatant reactions are found in individually occurring polymorphisms in the genes of the proinflammatory cytokines TNF-α, interleukin-1, and the anti-inflammatory counterpart IL-1RA [28].

### Secondary RANTES/CCL5 expression driven by TNF-α in FDOJ

**Fig. 10:**
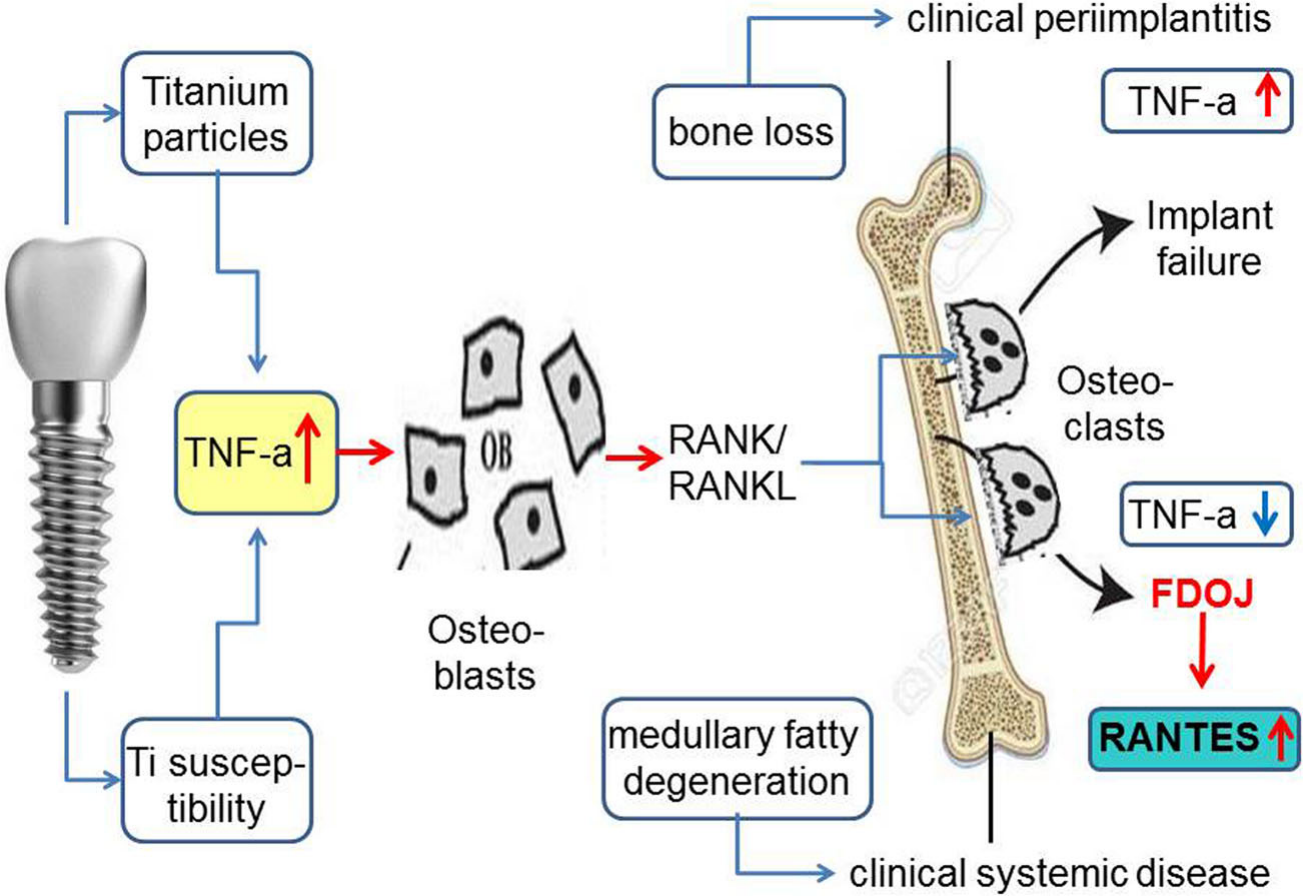
Possible side effects of titanium implants resulting either in bone loss and implant failure, (shown in the upper pathway), or in medullary fatty degenerative osteonecrosis with no implant loss but systemic interference by RANTES/CCL5 overexpression, (shown in the lower pathway)

At this point, the question arises as to whether there is an induced or synergistic interaction between the inflammatory TNF-α mediators secreted around T-IMPs and the highly overexpressed R/C levels found in our previous research. The inflammatory response of adipose tissue associated with a systemic inflammatory response is well known and widely discussed. Secretion of inflammatory cytokines mediates the systemic effects of adipose tissue inflammation. Most normal adult tissues contain few, if any, RANTES positive cells. In contrast, RANTES expression dramatically increases in inflammatory sites [29]. These results indicate a wider expression of RANTES than previously appreciated and suggest multiple physiologic roles for this soluble factor. In the case of dental implants, the human bone marrow is the effector tissue source in situ as the implant is located intramedullary. Studies show the ability of titanium wear particles in a human bone marrow cell culture to induce a significantly higher release of proinflammatory and osteolytic mediators which are responsible for the aseptic loosening of implants [4]. Beyond these findings based on TNF-α, our previous research points toward the next step of the process, namely the possibility of mediator cascades by R/C overexpression as found in FDOJ. Reduced blood flow and capillary density followed by ischemia in the medullary spaces of jawbone may lead to a hypoxic situation [30]. Adipocytes and the necrotic parts of fat cells are considered by many studies to be immunologically active. In understanding FDOJ, R/C, and ISD, the role of these immune effects is a relevant issue. While proinflammatory cytokines such as TNF-α and IL-6 are distributed early on during the acute stage of an injury or tissue infection, chemokines like R/C may be activated at a later time. They may play a crucial role in the transition of acute pain into a more chronic phenomenon. In conjunction with tissue damage or infection, ischemia-induced chemokine expression causes an increase in inflammatory cytokines [31]. R/C expression was spontaneous and continuous in most samples of mature adipocytes from omental and subcutaneous deposits and hypoxia and ischemia cause an approximately 36% increase in R/C. Human adipocytes express R/C and can thus be identified as a new cellular source of this messenger [32, 33]. The network of cytokine effects between TNF-α and the hyperactivated R/C cascades found in FDOJ (Fig. 10) has not yet been fully investigated in terms of the interactions, amplifications, and phases of action between the released cytokines in the tissues surrounding T-IMPs.

### RANTES/CCL5 overexpression in FDOJ and possible connection to systemic diseases

If the body does not succeed in correcting the metabolic disturbances in a FDOJ area of the jawbone within a certain period, increasing numbers of immune cells will be recruited from the fatty tissue. Proinflammatory signaling mediators such as R/C in particular affect the organism systemically and may result in chronic inflammatory processes or provoke further pathophysiological mechanisms. It is generally accepted that an imbalance between cytokines and their specific inhibitors is characteristic of chronic inflammatory conditions [34]. Cytokines merge to initiate an immune response and induce acute inflammatory events in the case of persistent chronic inflammation (CI). This means that in order to maintain healthy conditions, cytokine-producing mechanisms must be controlled [35]. FDOJ represents a new inflammatory cellular response phenomenon in that the cytokines are not triggered by the presence of bacteria or viruses. This is supported by the fact that levels of typical acute proinflammatory cytokines such as TNF-α and IL-6 are not found to increase in this process. As a matter of fact, these acute cytokines are found to be absent in the FDOJ samples. Accordingly, we have hypothesized that R/C signaling is a chronic disturbance that may contribute to the development of CI. The absence of acute inflammation in FDOJ denotes the subclinical and hidden proliferation of chronic immunological processes driven by R/C. R/C is a member of the CC chemokine family expressed in a wide array of immune and non-immune cells in response to stress signals. Because of the apparent dispensable nature of this molecule in normal physiology, R/C may represent an excellent therapeutic target in immunotherapy [36]. High levels of the inflammatory cytokine RANTES are found in the aging stem cell milieu [37]. Studies have shown that TNF-α stimulates the secretion of R/C in smooth muscle cells of the airways [38]. In the case of breast cancer, there is evidence of a synergistic osteoimmunological reaction to T-IMPs as TNF-α is an activator of the R/C promoter for mesenchymal stem cells in the tumor environment of breast cancer via its signal transduction pathway [39]. The TNF-α stimulation of the mesenchymal stem cells led to a dose-dependent increase in the expression of R/C in the tumor [39]. Research on R/C and rheumatoid arthritis shows the same mechanism: in non-stimulated synovial fibroblasts, the expression of mRNA was not detectable for R/C. However, as a result of stimulation with the monocytic cytokines TNF-α and IL-1beta, R/C increase was time and dose-dependent [40]. Volin found that in the case of rheumatoid arthritis in the synovial fluid with fibroblasts of the synovial tissue stimulated with IL-1b or TNF-α 40- to 50-fold higher R/C than in unstimulated tissue. R/C-activated chondrocyte functions are associated with joint inflammation and cartilage degradation in rheumatoid arthritis. IL-1b and TNF-α also induce the production of other cytokines including MCP-1/CCL2 and R/C, which are overexpressed in arthritic joints [41, 42]. R/C was also shown to be found in the human endometrium: Arima et al. investigated the effects of endometrial function modulators including lipopolysaccharides (LPS) TNF-α, IL-1b, IL-4, and IFN-g on R/C expression by endometrial stromal cells (ESC). The concentration of R/C in the culture of non-stimulated ESC was below the detection limit. The concentration of R/C was increased by the addition of TNF-α and IL-1b. Thus, transcription of R/C into ESC was stimulated by TNF-α and IL-1b in a dose-dependent manner [43]. Wolf et al. found that TNF-α induces the expression of the attractant chemokine R/C in cultured mesangial mouse cells. Fifty nanograms per milliliter recombinant TNF-α induces a significant increase in R/C signal transmission after 2 h. Also, IL-1b increased the expression of R/C mRNA [44]. The stimulatory chain from TNF-α and IL-1b to R/C can also have a pathogenomic effect in cardiovascular diseases. Activation of the endothelium is a critical moment in the inflammatory process and is associated with chemokine production in endothelial cells of human coronary arteries [45].

### Case report: titanium implants, R/C overexpression in jawbone, and ovarian cancer

**Fig. 11:**
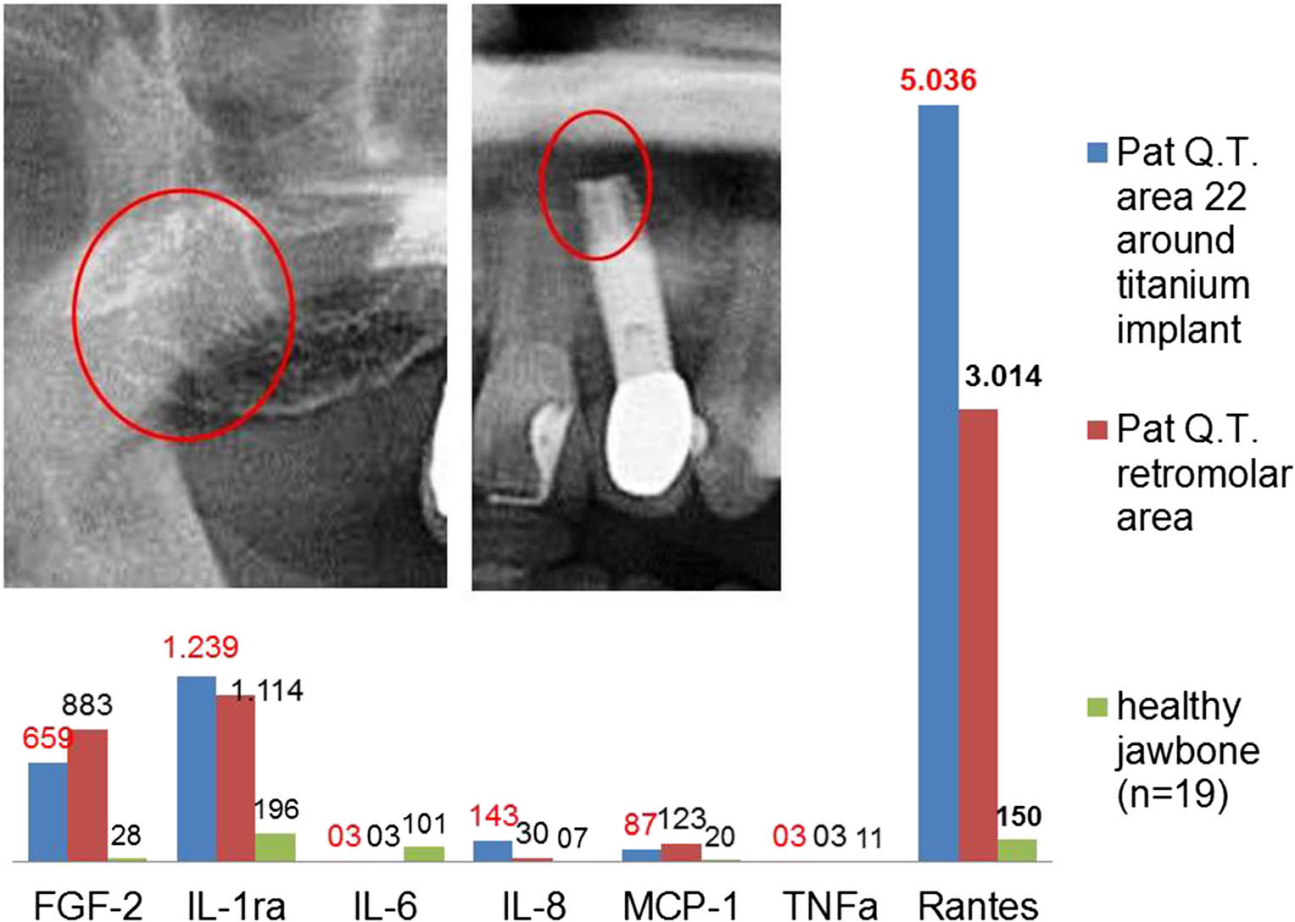
Radiological findings from a 2D-OPG of the inconspicuous retromolar area (left panel) and surrounding the titanium implant at area 22 (right panel). Graph shows osteoimmunology of retromolar FDOJ at area 18–19 (red column) compared to cytokine expression in the region surrounding the titanium implant at area 22 (blue column)

**Fig. 12:**
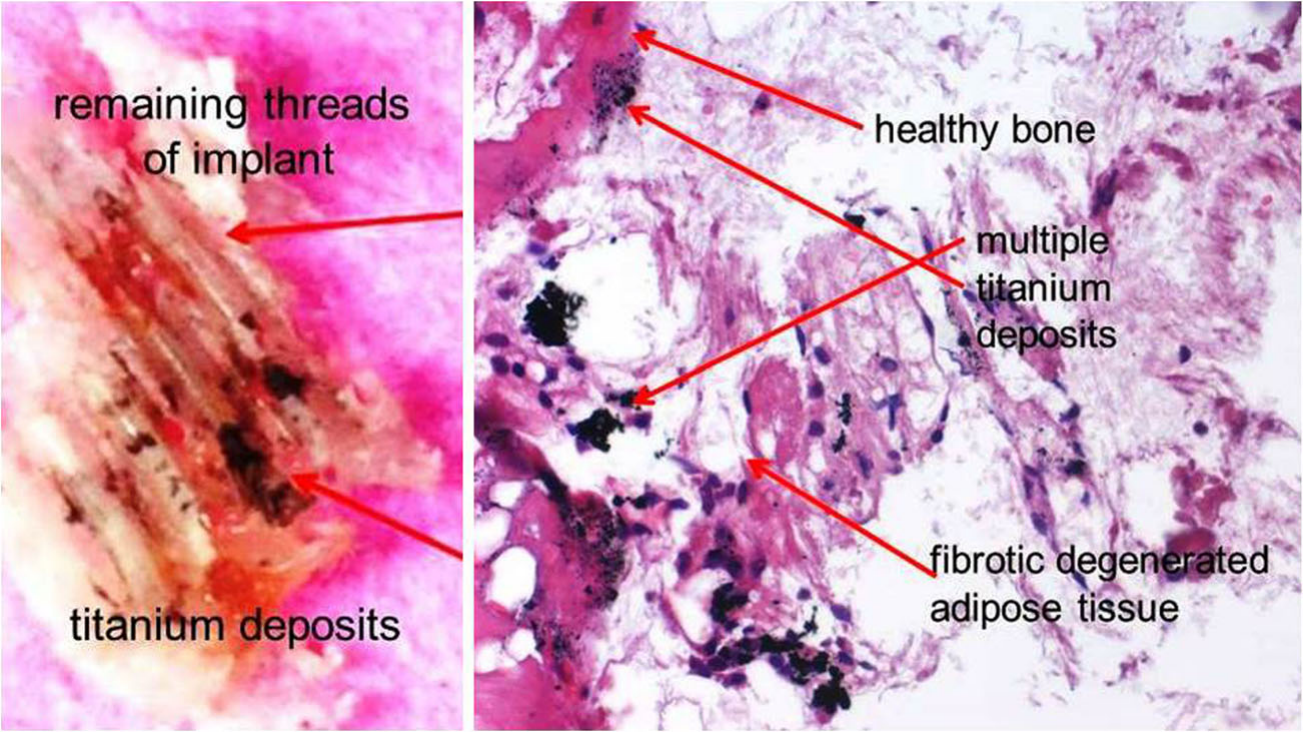
Clinical findings. Jawbone adjacent to titanium implant, area 17, with blackish metal in remaining threads caused by the implant (left panel). Titanium deposits on healthy bone and adjoining fibrotic, degenerated medullary adipose tissue with enlarged adipocytes (right panel)

A 52-year-old patient was suffering from an advanced OvC. The 2D-OPG of the red-edge retromolar areas and apical area of the T-IMP at tooth no. 22 showed no functional alterations of the jawbone. (See Fig. 11.) It was thus all the more surprising that the R/C expression in the vicinity of the T-IMP was almost twice as high as in the retromolar FDOJ area. In addition, the acute cytokines TNF-α and IL-6 were negligible in the two FDOJ areas and were, in fact, at levels lower than the values of a standard sample. The relationship between R/C and OvC is evident in the literature: plasma values of R/C are higher in OvC patients compared to the reference and control groups. Women with OvC have significantly higher R/C levels in the peritoneal fluid than patients with undifferentiated carcinoma [45]. With these connections between R/C and OvC in mind, we found the retromolar FDOJ area had a 20-fold increased R/C value, while the overexpression in the area surrounding the T-IMP was 33-fold. The levels in T-IMP adjacent jawbone were almost double the R/C signaling effect on OvC in comparison to the retromolar FDOJ area. The histological examination report of the curetted FDOJ area surrounding the T-IMP at area 22 included the following findings: “… marrow areas with stromafibrosis and proliferated capillaries and also fibrillar degeneration of the cytoplasma, with trophic disturbances and non-active osteitis. No osteomyelitis.” Figure 12 shows the microscopic image of area 22 with titanium deposits found on healthy bone adjacent to fibrous and fatty degenerated medullary adipose tissue with enlarged adipocytes which is typical of FDOJ.

### Implantation and possible cytokine cascades—different phases during implant insertion and integration

Perala demonstrated the induction of TNF-α in vitro after coincubation of native implant material which ensures that immunogenic particles are released from the materials [46]. Concerning cytokine expression in the context of an implant and the associated phases of healing, the analysis during different stages of implantation reveals several new phases of cytokine-triggered signaling pathways: (a) The T-IMP is placed in an ischemic area of subclinical FDOJ due to the radiographically inconspicuous nature of FDOJ and the absence of alternative methods of measuring bone density. The systemic effect hitherto is subclinical and therefore free of symptoms. (b) The acute wound setting initiated by the insertion of an implant in which the surgical trauma induces the release of acute cytokines creating inflammatory cascades via TNF-α, Il-6, and Il-1b expression. (c) TI particles provoke expression of TNF-α at a later stage of wound healing. (d) In the medium to long term, TNF-α expression provokes increased secretion of R/C.

**Fig. 13:**
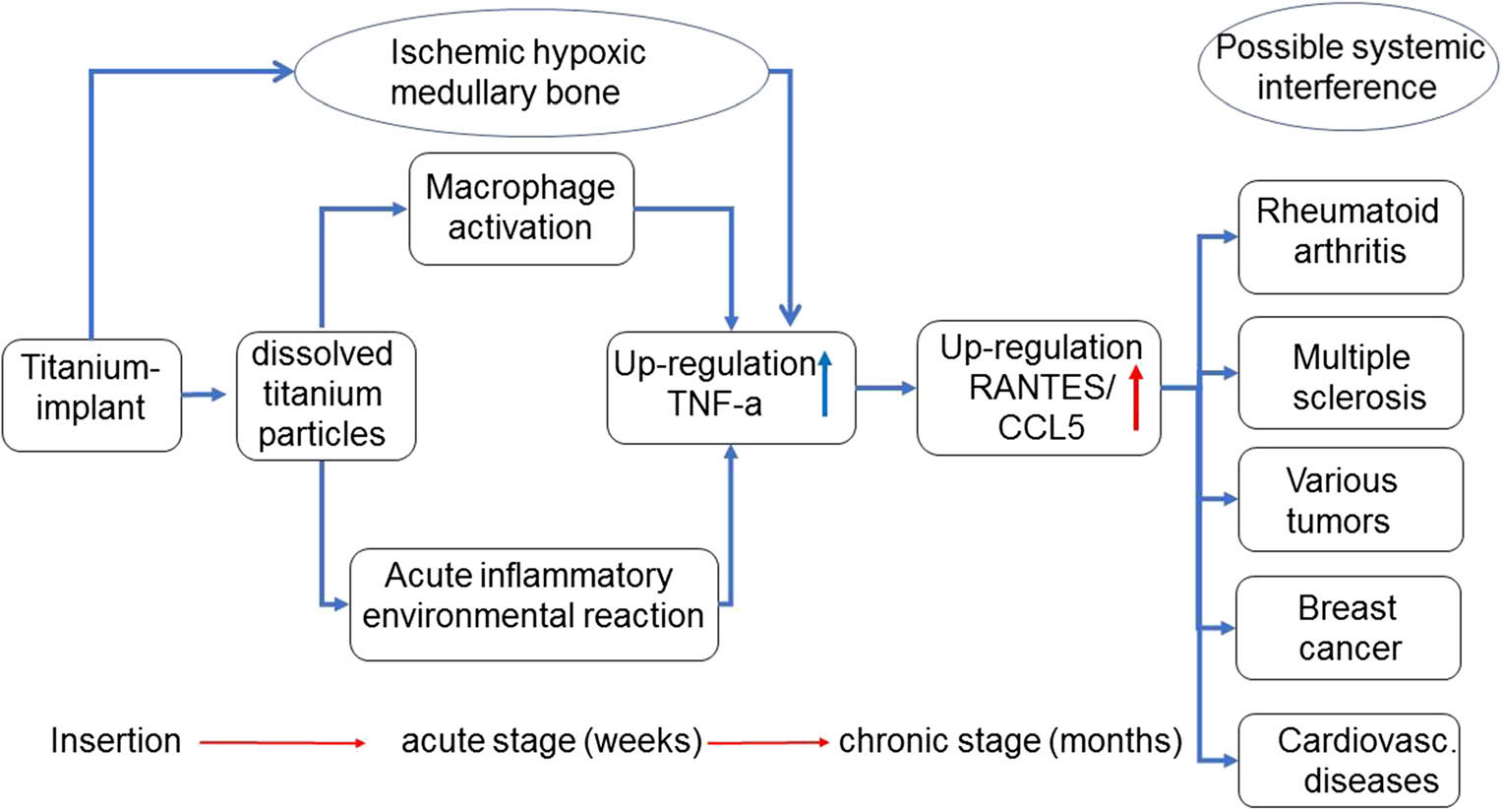
Cytokine pathways in the implant area during osseointegration from TNF-α to R/C overexpression with long-term, negative immune response, and effects

The problems for the clinician in this context include the following: (a) the clinical stability of the T-IMP leads to the misdiagnosis of an apparently inflammation-free osseointegration,( b) the radiological and clinical inconspicuousness of T-IMP, and (c) the systemic symptoms of an ISD are not directly related to the T-IMP because they occur only after a certain amount of time. As a result, an osteoimmunological scenario in the case of implantation is conceivable, as shown in Fig. 13.

The process described in Fig. 13 confirms the findings of basic research: while proinflammatory cytokines such as TNF-α and IL-6 are released early in the acute stage of injury or tissue infection, there is considerable evidence that chemokine expression, such as R/C, may occur at a later stage. In turn, this provokes a transition from acute inflammation to a chronic state of “silent inflammation.” Accordingly, a purely clinical and symptom-focused assessment of T-IMPs is insufficient. X-rays also fail to indicate the derailed mediator process (cytokines, interleukins) triggered by T-IMPs. Consequently, the evaluation and indication of T-IMPs must also be viewed from a systemic perspective according to a personalized and individually tailored approach. Following the recommendations of Predictive Preventive Personalized Medicine (PPPM), individually targeted prevention is a crucial aspect of this therapeutic concept. In failing to recognize this, detrimental local and systemic health consequences may occur in the host that are concealed by the apparent success of a “stable implant.”

## Conclusions

Our data from this preliminary investigation provides some evidence for the immunological relationship between T-IMPs and FDOJ. As far as we are aware, this study is the first to clinically describe the possible connection between T-IMPs and FDOJ as a vector or cause of the so-called silent inflammation. Although the extent to which increased expression of R/C derived from FDOJ sites contributes to immune-mediated diseases is unknown, the results described herein provide evidence for the possible interaction between T-IMPs, R/C signaling, and general health. A comprehensive understanding of the complex networks outlined in this paper requires further research into the fundamental physical, chemical, and electrical properties of T-IMPs which have a causal role with respect to the expression of R/C cytokines and subsequent symptoms of “silent inflammation.” The challenge posed by these discoveries is the need to raise awareness of the potentially critical interplay between T-IMPs and FDOJ throughout the medical and dental communities. Removal of T-IMPs and surgical debridement of surrounding FDOJ areas may diminish R/C overexpressed signaling pathways and thus possibly reduce inflammatory input and associated symptoms. This research is especially relevant to the objectives of PPPM [47] given that, in certain individuals, a particularly subtle and chronic inflammatory process surrounding T-IMPL may occur and, in turn, contribute to a derailed immune system. Depending on individual genetic and environmental conditions, FDOJ surrounding T-IMPL should be given further consideration where appropriate, leading doctors and dentists to a broader interest in preventive therapies. It is recommended that the results presented herein are used to guide further research, in particular the demonstrated measuring of inflammatory cytokines found in bone tissue, which may provide a promising step toward further multidisciplinary considerations for personalized dentistry and prevention [48]. The results submitted by the authors highlight the need for a large multi-center prospective study with clearly defined cohorts in order to reliably clarify the causative pathways involved.

## Abbreviations

CFS: Chronic fatigue syndrome
CI: Chronic inflammation
FDOJ: Fatty degenerative osteonecrosis of the jaw
FDOJ-T-IMP: Fatty degenerative osteonecrosis of the jaw adjacent to a titanium implant
HJB: Healthy jawbone
ISD: Immune system disorders
MS: Multiple sclerosis
OvC: Ovarian cancer
PPPM: Predictive Preventive Personalized Medicine
R/C: RANTES/CCL5
RA: Rheumatic arthritis
TAU: Transalveolar ultrasound technology
TI: Titanium
T-IMP: Titanium implant
TNF-α: Tumor necrosis factor-alpha
TrN: Atypical facial pain/trigeminal neuralgia
